# Fate‐mapping of erythropoietin‐producing cells in mouse models of hypoxaemia and renal tissue remodelling reveals repeated recruitment and persistent functionality

**DOI:** 10.1111/apha.13768

**Published:** 2022-01-16

**Authors:** Sophie L. Dahl, Svende Pfundstein, Rico Hunkeler, Xingtong Dong, Thomas Knöpfel, Patrick Spielmann, Carsten C. Scholz, Karen A. Nolan, Roland H. Wenger

**Affiliations:** ^1^ Institute of Physiology University of Zurich Zurich Switzerland; ^2^ National Center of Competence in Research “Kidney.CH” Zurich Switzerland

**Keywords:** chronic kidney disease, erythropoietin, hypoxia, PHD inhibitor, renal anaemia, tissue fibrosis

## Abstract

**Aim:**

Fibroblast‐like renal erythropoietin (Epo) producing (REP) cells of the corticomedullary border region “sense” a decrease in blood oxygen content following anaemia or hypoxaemia. Burst‐like transcription of Epo during tissue hypoxia is transient and is lost during fibrotic tissue remodelling, as observed in chronic kidney disease. The reason for this loss of Epo expression is under debate. Therefore, we tested the hypothesis that REP cell migration, loss and/or differentiation may cause Epo inhibition.

**Methods:**

Using a reporter mouse that allows permanent labelling of active REP cells at any given time point, we analysed the spatiotemporal fate of REP cells following their initial hypoxic recruitment in models of hypoxaemia and renal tissue remodelling.

**Results:**

In long‐term tracing experiments, tagged REP reporter cells neither died, proliferated, migrated nor transdifferentiated into myofibroblasts. Approximately 60% of tagged cells re‐expressed Epo upon a second hypoxic stimulus. In an unilateral model of tissue remodelling, tagged cells proliferated and ceased to produce Epo before a detectable increase in myofibroblast markers. Treatment with a hypoxia‐inducible factor (HIF) stabilizing agent (FG‐4592/roxadustat) re‐induced Epo expression in the previously active REP cells of the damaged kidney to a similar extent as in the contralateral healthy kidney.

**Conclusions:**

Rather than cell death or differentiation, these results suggest cell‐intrinsic transient inhibition of Epo transcription: following long‐term dormancy, REP cells can repeatedly be recruited by tissue hypoxia, and during myofibrotic tissue remodelling, dormant REP cells are efficiently rescued by a pharmaceutic HIF stabilizer, demonstrating persistent REP cell functionality even during phases of Epo suppression.

## 1 INTRODUCTION

Circulating erythropoietin (Epo) is mainly produced by poorly defined interstitial cells of the kidney.^1^ Upon a decrease in blood oxygen concentration, renal tissue hypoxia leads to transient transcriptional bursts in renal Epo‐producing (REP) cells.^2^, ^3^ The factors that determine the selection of actively Epo‐expressing “on” cells from the large pool of dormant “off” REP cells are currently unknown.^4^, ^5^ While no specific marker (except Epo itself) has been reported to unambiguously identify REP cells, recent work demonstrated that REP cells express markers common to fibroblasts, pericytes, mesenchymal stem cell‐like cells and even neurons.^6^, ^7^ For instance, Epo mRNA has been detected in spatially distinct sub‐populations of PDGFRβ positive cells, characterized by the exclusive co‐expression of either CD73 (*NT5E*), Gli1, tenascin C or SMMHC,^8^ suggesting that REP cells can be recruited from a pool of mesenchymal cells with diverse differentiation phenotypes. It has been suggested that REP‐to‐myofibroblast transdifferentiation, as shown by increased levels of αSMA (*ACTA2*) protein, goes along with the permanent loss of Epo expression,^9^, ^10^ which appears to be consolidated by epigenetic modification of the *EPO* locus.^11^, ^12^

REP cells are predominantly located within the corticomedullary border region.^6^, ^7^, ^13^, ^14^ This region overlaps with a steep drop in oxygen bioavailability,^7^ suggesting that the spatial distribution of determined but dormant “off” REP cells is mainly based on the oxygen partial pressure of their microenvironment.^3^ Under severe anaemic conditions virtually all interstitial fibroblast‐like cells of the renal cortex (including outer cortex) and medulla can principally be recruited for Epo production.^15^ However, it is currently poorly understood how a small subset of REP cells is recruited from this large pool of cells to undergo transcriptional Epo bursts following hypoxic or pharmaceutical activation.

During chronic kidney disease (CKD), Epo‐deficiency anaemia is commonly observed and needs to be treated by recombinant Epo injections or, more recently, by oral application of hypoxia‐inducible factor (HIF) stabilizers.^16^ Of the five drugs that have recently been clinically approved,^17^ roxadustat (FG‐4592) was the first compound authorized for the treatment of renal anaemia. However, it is neither known why exactly Epo expression is lost during CKD nor which cells are recruited by roxadustat in the diseased kidney. Current hypotheses center around renal tissue hyperoxia due to decreased tubular oxygen consumption,^3^, ^18^, ^19^ TGFβ signalling,^20^ and/or inflammatory REP‐to‐myofibroblast transdifferentiation^4^, ^5^ as possible causes of suppressed or lost Epo expression. While the known rapid induction of Epo by roxadustat^21^, ^22^ would be consistent with a counteraction of tissue hyperoxia,^23^, ^24^ it is currently unclear how HIF stabilizers reverse epigenetically fixed myofibroblast transdifferentiation.

Genetically modified mouse models to permanently label and trace REP cells during physiological adaptation, differentiation and disease progression would principally allow for the investigation of the transient nature of Epo expression and of the recruitment of REP cells in models of CKD. However, the previously available mouse models either used non‐*Epo*‐derived regulatory DNA elements of marker genes and hence did not exclusively label REP cells,^11^ or they used *Epo*‐derived regulatory elements but non‐conditionally labelled REP cells at non‐defined time points during kidney development and growth.^15^

To test the hypothesis that REP cell migration, loss and/or differentiation may cause Epo inhibition, we recently generated a new mouse model that allows for the permanent, conditional and exclusive labelling of currently active “on” REP cells.^7^ Using this mouse model, we investigated the long‐term fate of previously active REP cells in the healthy and diseased kidney. Unexpectedly, myofibroblast transdifferentiation was not the major cause of the initial Epo loss during a unilateral model of renal tissue remodelling as observed during CKD, and roxadustat efficiently restored Epo expression in the silenced REP cells of the diseased kidney.

## 2 RESULTS

### 2.1 Transient induction of Epo despite ongoing hypoxia

**FIGURE 1:**
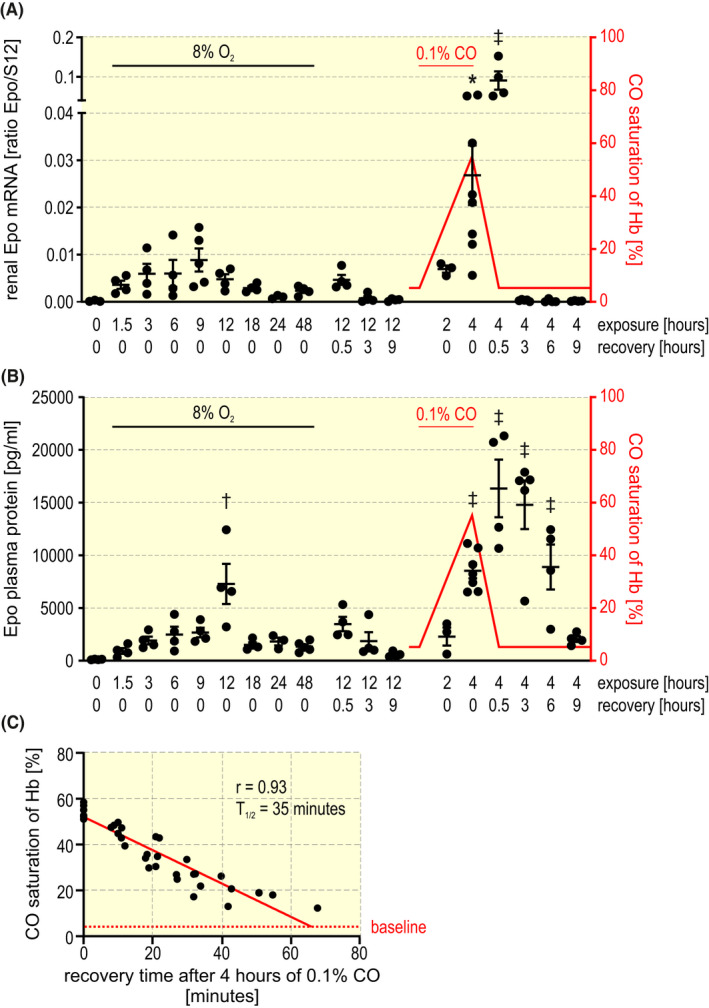
Kinetics of Epo production under hypoxic conditions. Mice were exposed to inspiratory hypoxia (8% O_2_) or carbon monoxide (0.1% CO in air) for the indicated time periods. Normoxic recovery experiments were started after 12 hours 8% O_2_ or 4 hours 0.1% CO exposure for the indicated durations. A, Kidney Epo mRNA levels were determined by RT‐qPCR and are displayed relative to the ribosomal protein S12 mRNA levels. B, Epo plasma protein levels were determined by ELISA. Haemoglobin (Hb) CO saturation is indicated in red. One‐way ANOVA followed by Dunnett’s post‐hoc correction was used to statistically evaluate Epo induction versus the normoxic (20.9% inspiratory O_2_) control animals (average ± SEM; *P < .05, ^†^ *P* < .01, ^‡^ *P* < .001). C, Mice were exposed for 4 hours to 0.1% CO, resulting in 54.8 ± 2.6% (n = 7) CO saturation of haemoglobin. Following the return to normal air inspiration, CO saturation of haemoglobin was spectroscopically measured at the indicated time points. Regression analysis suggests a linear decline of carbon monoxide saturation of haemoglobin (n = 7). The baseline of 4.57 ± 2.03% CO saturation of haemoglobin (n = 7) is indicated by the dashed red line

In addition to the conditionally induced nuclear translocation of Cre^ERT2^ by tamoxifen, transgene expression in *Epo‐Cre^ERT2^* mice required hypoxic activation to label a sufficient number of REP reporter cells. For time‐resolved REP cell fate studies it was important to define the Epo induction kinetics. Therefore, mice were exposed to normobaric hypoxia (8% O_2_) or carbon monoxide (0.1% CO) (Figure 1). Consistent with previous reports in rats,^25^, ^26^ a transient induction of Epo mRNA preceded the circulating Epo protein induction (Figure 1A,B, respectively). CO haemoglobin saturation reached ~55% after 4 hours and linearly declined with a half‐life of ~35 minutes (Figure 1C). Compared to 8% O_2_ a brief 4 hours 0.1% CO exposure resulted in a much stronger and temporally more defined transient increase in Epo expression. Therefore, this hypoxic stimulus was used for conditional REP cell tagging following tamoxifen administration. The cellular Epo mRNA expression heterogeneity was assessed after 4 hours 0.1% CO using single‐molecule fluorescence in situ hybridization (FISH) (Figure S1A). According to the cumulative frequency distribution shown in Figure S1B, ~9.4% of all cells account for ~29% of total Epo mRNA (high‐contributors) whereas ~62% of all cells account for only ~26% of total Epo mRNA (low‐contributors).

### 2.2 Long‐term survival of resident REP cells

**FIGURE 2:**
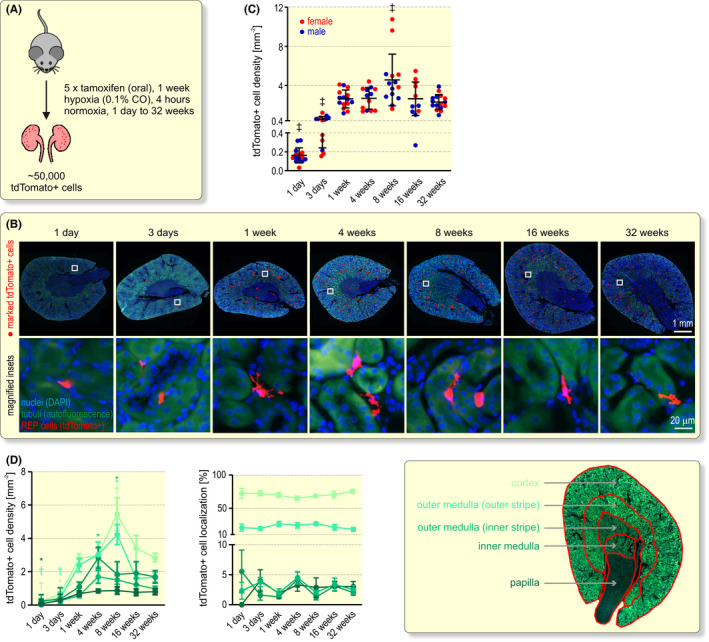
Long‐term fate of renal Epo‐producing (REP) cells. A, Schematic illustrating the conditional tagging of REP reporter cells by constitutive red fluorescent tdTomato expression in *Epo‐Cre^ERT2^#1*x*tdTomato* mice. Following exposure to tamoxifen and a brief hypoxic stimulus, tdTomato^+^ cells were analysed 1 day to 32 weeks later. B, Detection of tdTomato^+^ REP reporter cells. Nuclei were stained with DAPI (blue) and tubuli were visualized by their autofluorescence (green). REP cells are marked by red dots (upper panel) and the endogenous red tdTomato fluorescence is shown in the magnified square insets (lower panel). C, Quantification of tdTomato^+^ REP cell density. Each data point represents the average value of 4 to 8 kidney slices derived from one mouse. Shown is the average per group ± SD. D, Spatial quantification of tdTomato^+^ REP reporter cells in the cortex, outer and inner segments of the outer medulla, inner medulla and papilla. At least 750 tdTomato^+^ cells were analysed per time point and are shown as cell density (left panel) and percentage distribution (middle panel). The right panel illustrates the manual alignment of the kidney regions based on the endogenous green tubular autofluorescence. C and D, One‐way ANOVA followed by Dunnett’s post‐hoc correction was used to statistically evaluate tdTomato^+^ REP cell changes versus the corresponding values at 1 week (*P < .05, ^†^ *P* < .01, ^‡^ *P* < .001)

Potential explanations for the transient Epo induction include REP cell loss and/or REP cell migration to areas with a less hypoxic microenvironment. To investigate these possibilities, we permanently tagged REP cells in 2 to 3 months old mice by a transient combined tamoxifen/CO stimulus and analysed the REP reporter cells 1 day to 32 weeks later (Figure 2A). Using fluorescence microscopy of whole kidney slices, tagged REP cells were detected exclusively in the peritubular interstitial compartment (Figure 2B). Automated detection and counting revealed that the number of tagged REP cells increased during the first 3 days after induction and roughly remained constant for the following 1 to 32 weeks, except for two outliers at 8 weeks (Figure 2C). After ensuring that the REP cell density remained constant throughout all horizontal planes of the kidney (Figure S2), a total of ~50 000 tagged REP reporter cells per mouse was estimated, which likely reflects the high contributors shown in Figure S1. There was a trend towards higher REP cell numbers in female mice which usually did not reach significance (except at 8 and 16 weeks; *P* < .01, two‐way ANOVA followed by Bonferroni’s post‐hoc correction). The delayed increase in the number of tagged cells during the first week might at least partially be explained by the different mRNA kinetics of Epo compared with Cre^ERT2^, as observed in some of the mice (Figure S3), but also tdTomato protein accumulation may be different from Epo kinetics. Tagged REP cells were mainly detected within the corticomedullary border region but there were also isolated REP cells in the inner regions of the kidney. Normalized REP cell densities in these diverse kidney compartments remained strikingly constant over 32 weeks (Figure 2D), suggesting that REP cells likely neither die nor migrate and that the decrease in Epo expression must therefore be based on a cell‐intrinsic process. To exclude a theoretically possible high turnover of these cells, we stained for the Ki67 proliferation marker, but detected only sporadic Ki67^+^ cells with virtually no overlap with tagged REP cells (Figure S4).

### 2.3 Aged REP cells do not transdifferentiate and can repeatedly be recruited for Epo production

**FIGURE 3:**
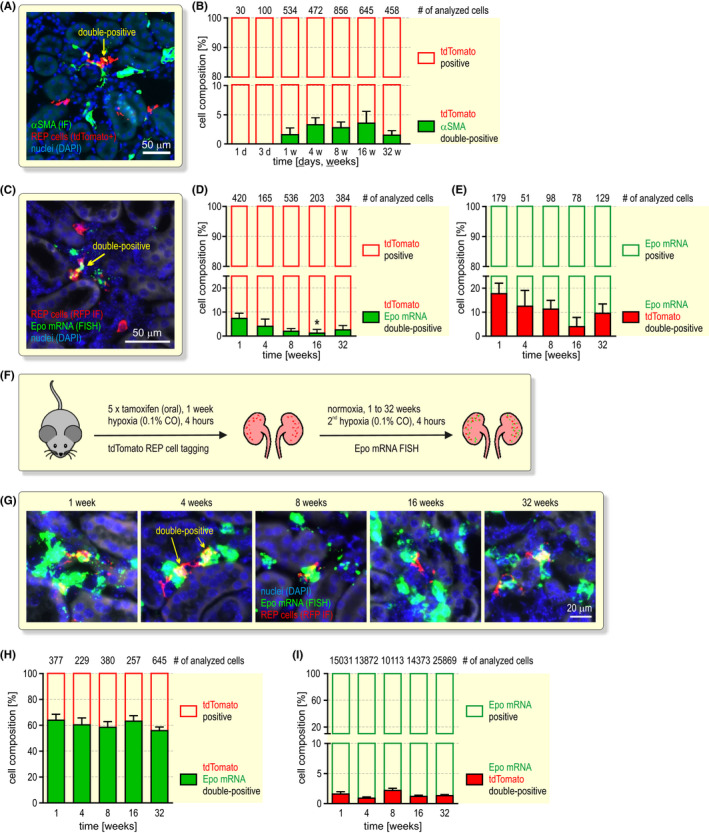
Transdifferentiation and recruitment of ageing REP cells. A, Example of αSMA immunofluorescence (IF; green) combined with REP cell tagging in *Epo‐Cre^ERT2^#1*x*tdTomato* mice (red) and nuclear staining by DAPI (blue). B, Kidneys were excised at the indicated time points after the 4 hours 0.1% CO stimulus and the labelled reporter cells were automatically counted. The number of analysed cells derived from 8 mice per timepoint is indicated. C, Example of Epo mRNA fluorescence in situ hybridization (FISH; green) combined with immunofluorescence detection of tdTomato in tagged REP cells by an anti‐red fluorescent protein (RFP) antibody (red) and nuclear staining by DAPI (blue). Epo mRNA/tdTomato double‐positive REP cells are depicted in yellow. D and E, Quantification of tdTomato/Epo mRNA double‐positive cells. The numbers of analysed cells derived from 5 to 8 mice per timepoint are indicated. F, Schematic illustrating the conditional tagging of REP reporter cells followed by a second hypoxic stimulus after the indicated time periods. G, Detection of Epo mRNA/tdTomato double‐positive REP cells as in (C). H and I, Quantification of tdTomato/Epo mRNA double‐positive cells. The numbers of analysed cells derived from 5 to 11 mice per timepoint are indicated. B, D, E, H, I, All data are shown as average + SEM. One‐way ANOVA followed by Dunnett’s post‐hoc correction versus the corresponding values of week 1 was used to statistically evaluate cell compositions (*P < .05)

A common explanation for a decline in Epo expression is REP‐to‐myofibroblast transdifferentiation.^4^, ^12^ We hence used αSMA to detect myofibroblast transdifferentiation of tagged REP reporter cells (Figure 3A). Among the tdTomato^+^ cells, αSMA^+^ double‐positive cells were undetectable during the first 3 days and remained very low (1.5% to 3.6%) during the 32 weeks observation period (Figure 3B), suggesting that REP cells do not generally transdifferentiate once they were hypoxically stimulated and expressed Epo. These findings raised the question whether ageing REP cells still express Epo. Therefore, we used highly sensitive Epo mRNA single‐molecule FISH to detect even weakly (ie below the activity required to tag REP cells) active REP cells under normoxic conditions (Figure 3C). While most of the tagged REP cells did not express Epo mRNA anymore, a small proportion (7.8% after 1 week to 2.6% after 32 weeks) of tagged REP cells was Epo mRNA positive and hence still contributed to the low normoxic Epo production (Figure 3D). *Vice versa*, 18.2% (1 week) to 9.9% (32 weeks) of all Epo mRNA positive cells belonged to the previously active tagged REP reporter cells (Figure 3E).

We next assessed whether a repeated hypoxic stimulus could again recruit the previously active (ie tdTomato^+^) cells from the dormant “off” REP cell pool. Following a second identical CO exposure (without tamoxifen or recovery period), Epo mRNA was detected by FISH (Figure 3F). This procedure resulted in a strong acute induction in Epo mRNA (Figure 3G) compared to the normoxic experiments shown above. At all time points (1 to 32 weeks) after the first exposure, ~60% of the tdTomato^+^ REP reporter cells were able to re‐express Epo, without any decline over time (Figure 3H). Of the large number of Epo mRNA^+^ REP cells detected by highly sensitive FISH (~1200 cells per slice), ~1.5% corresponded to tdTomato^+^ REP reporter cells (Figure 3I), confirming that only REP cells undergoing a strong transcriptional Epo burst were tagged during the initial brief CO exposure. In summary, these findings suggest the existence of a large and long‐lived dormant pool of resident REP cells. A hypoxic insult stochastically recruits a subset of these cells with no apparent preference for the previously most active cells.

### 2.4 Pharmaceutical Epo induction recruits dormant REP cells from the same spatially defined area as hypoxia

**FIGURE 4:**
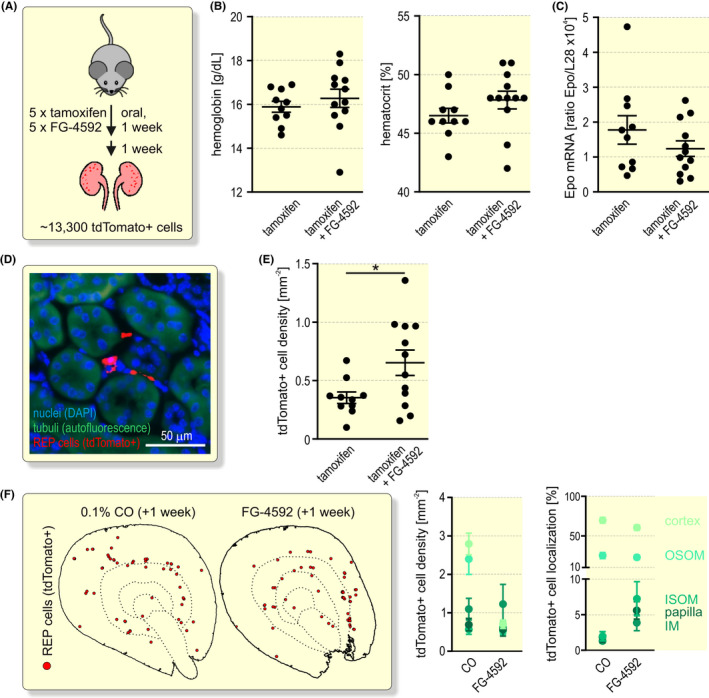
Pharmaceutical Epo induction by FG‐4592 (roxadustat). A, Schematic illustrating the conditional tagging of REP reporter cells by a combined gavage of 5 doses of tamoxifen alone or together with FG‐4592. Mice were analysed 1 week after the last dose. B, Haemoglobin and haematocrit measurement by whole‐blood spectroscopy of venous blood samples. C, Kidney Epo mRNA levels were determined by RT‐qPCR and are displayed relative to the ribosomal protein L28 mRNA levels. D, Detection of tdTomato^+^ REP cells. Tubuli were visualized by their autofluorescence (green) and nuclei were stained with DAPI (blue). E, Quantification of tdTomato^+^ REP cell density. Each data point represents the average value of 4 kidney slices derived from one mouse. B, C, E, Shown is the average per group ± SEM. Student’s unpaired *t* test were used to statistically evaluate changes versus the tamoxifen‐treated controls (*P < .05). F, Spatial distribution of tdTomato^+^ REP reporter cells. Shown are marked cells from 1 or 4 consecutive kidney sections one week after treatment with CO or FG‐4592, respectively (left panel). Automated quantification of tdTomato^+^ REP cells in the cortex, outer and inner segments of the outer medulla (OSOM and ISOM, respectively), inner medulla (IM) and papilla. In total, 660 tdTomato^+^ cells of 12 FG‐4592 treated mice were analysed and are shown as cell density and percentage distribution (right panel). The data of the CO‐exposed mice were duplicated from Figure 2

Because an oral Epo‐inducing drug is likely more uniformly distributed in the kidney than oxygen, which is variably consumed along the renal tubule, it could be assumed that pharmaceutical PHD inhibition activates REP cells in a more widely distributed area of the kidney than hypoxia. To test this hypothesis, FG‐4592 (roxadustat) was simultaneously applied together with tamoxifen to conditionally induce tdTomato in *Epo‐Cre^ERT2^#1*x*tdTomato* mice (Figure 4A). FG‐4592 treatment tagged ~13 300 REP cells per mouse, corresponding to ~27% of the REP cells tagged following CO exposure. One week after the last dose, FG‐4592 mildly induced haemoglobin and haematocrit, albeit with a few non‐responders (Figure 4B). Kidney Epo mRNA was no longer elevated one week after treatment (Figure 4C). Consistent with the rather weak response to FG‐4592, fluorescence microscopy of kidney slices (Figure 4D) revealed a significant ~1.8‐fold induction of the number of tdTomato^+^ REP reporter cells (Figure 4E). Interestingly, these cells were also mainly located in the inner cortical and outer medullary regions (Figure 4F), that is, showed a distribution that was similar to the hypoxia experiments shown in Figure 2.

### 2.5 Upon kidney damage, REP cells proliferate but cease to produce Epo

**FIGURE 5:**
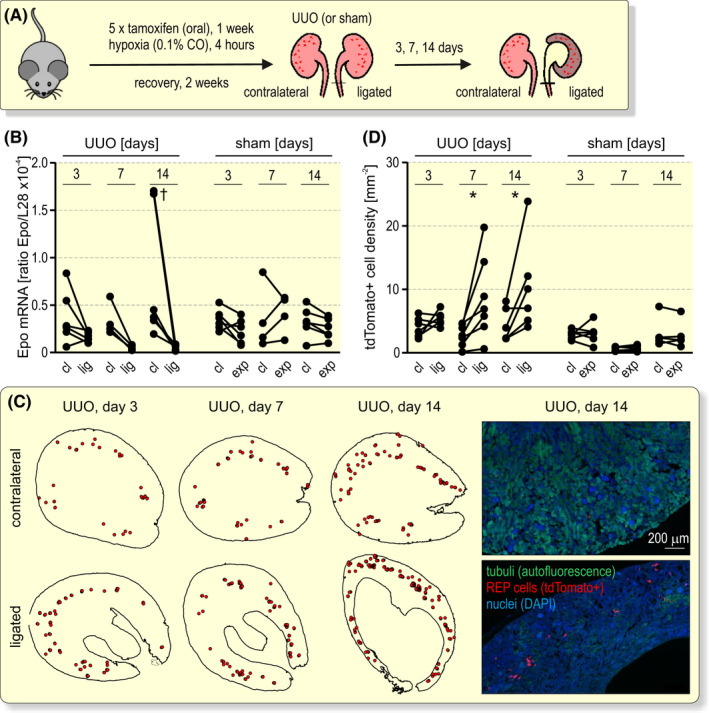
REP cell fate during fibrotic tissue remodelling. A, Schematic illustrating unilateral ureteral obstruction (UUO) 2 weeks after the conditional tagging of REP reporter cells. B, Kidney Epo mRNA levels were determined by RT‐qPCR and are displayed relative to the ribosomal protein L28 mRNA levels. Paired data from the contralateral (cl) and ligated (lig) or exposed (exp) kidneys of the UUO or sham surgeries, respectively, are shown. C, Distribution of tdTomato^+^ REP reporter cells. Shown are marked cells from one representative kidney section (left panel). Representative fluorescence microscopy pictures are shown in the right panel. D, Quantification of tdTomato^+^ REP cell density. Each data point represents the average value of 4 to 8 kidney slices derived from one mouse. Because of the loss of medullary tissue during UUO, this analysis focused on the cortex. B and D, Repeated measures two‐way ANOVA followed by Bonferroni’s post‐hoc correction was used to statistically evaluate Epo mRNA and tdTomato^+^ REP cell changes in ligated or exposed versus contralateral kidneys (*P < .05, ^†^ *P* < .01)

CKD generally results in the loss of Epo production and renal anaemia. To investigate whether REP cell death is involved in this phenomenon, we used unilateral ureteral obstruction (UUO) as a mouse model for fibrotic tissue remodelling during CKD in the ligated kidney while maintaining systemic kidney function, including circulating Epo levels, by the contralateral kidney (Figure 5A). As shown in Figure 5B, Epo mRNA strongly dropped after 3 days and was at baseline after 7 and 14 days in all ligated but not sham‐operated kidneys compared with the contralateral kidneys. To study the fate of recently active REP cells, we conditionally tagged them and started UUO 2 weeks later (Figure 5A). Consistent with a previous report on REP cell proliferation during UUO,^27^ far more tdTomato^+^ REP reporter cells were observed in the ligated than in the contralateral kidneys while tubular autofluorescence vanished (Figure 5C). Automated detection and counting revealed that the number of reporter cells increased by 1.5 to 5‐fold in 12 of 13 (1 kidney remained unchanged) ligated kidneys investigated at UUO days 7 and 14. Consistent with the Epo mRNA levels, no difference in REP reporter cell number was observed between sham‐operated and contralateral kidneys (Figure 5D). These results rule out that REP cell loss is responsible for the concomitant decrease of Epo mRNA expression during kidney disease progression.

### 2.6 Loss of Epo expression during kidney disease progression precedes REP cell transdifferentiation

**FIGURE 6:**
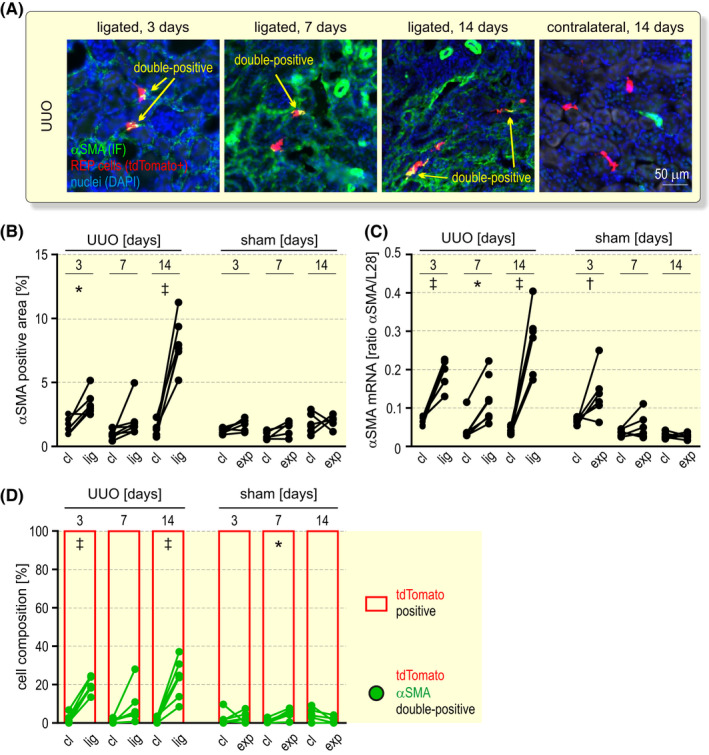
Transdifferentiation analysis of REP cells during fibrotic tissue remodelling. A, αSMA immunofluorescence (IF; green) after 3 to 14 days unilateral ureteral obstruction (UUO) as outlined in Figure 5A. REP reporter cells were tagged by tdTomato (red) and nuclei were stained with DAPI (blue). Shown are exemplary areas containing tdTomato/αSMA double‐positive cells (yellow). B, Automated quantification of the αSMA^+^ area in the contralateral (cl) and ligated (lig) or sham‐operated exposed (exp) kidneys. C, Kidney αSMA mRNA levels were determined by RT‐qPCR and are displayed relative to the ribosomal protein L28 mRNA levels. D, tdTomato positive (red) and tdTomato/αSMA double‐positive (green) cell composition. Labelled cells were automatically counted and the values of at least 800 cells per timepoint are shown. B‐D, Repeated measures two‐way ANOVA followed by Bonferroni’s post‐hoc correction was used to statistically evaluate αSMA changes in ligated or exposed versus contralateral kidneys (*P < .05, ^†^ *P* < .01, ^‡^ *P* < .001)

In a previous study using a UUO mouse model with a reporter gene knock‐in into the *Epo* locus, 50% and 80% of REP cells have been found to become αSMA positive as soon as after 2 and 3 days, respectively, of ureter ligation.^27^ While these results suggested myofibroblast transdifferentiation as the main cause of the loss of Epo expression during CKD, REP cells had been activated and tagged by a genetic background of severe congenital anaemia, limiting the relevance of this model for the investigation of renal anaemia. Therefore, we analysed αSMA expression at 3, 7 and 14 days of UUO in REP reporter cells that were tagged only 2 weeks before the onset of UUO (Figure 6A). Both, the αSMA positive area (Figure 6B) as well as αSMA mRNA levels (Figure 6C) strongly increased at all time points of UUO in the ligated kidneys when compared to the contralateral kidneys of the same mice, confirming progressive myofibrosis. While this αSMA increase was absent in sham‐operated kidneys on the protein level, some mice showed an induction on the mRNA level at early time points after the operation. Surprisingly, automated quantification of tdTomato^+^ REP reporter cells revealed that only a minority (maximal average of 23%, reached 14 days after ureter ligation) also expressed αSMA (Figure 6D).

To confirm this unexpected result, we additionally stained the kidney sections of the operated mice for the mesenchymal intermediate filament vimentin and the extracellular matrix protein collagen‐1, two markers whose enhanced expression is associated with kidney fibrosis. As expected, vimentin protein and mRNA strongly increased during UUO (Figure S5A‐C). On average, 16% and 40% of the tdTomato^+^ REP cells co‐expressed vimentin in the contralateral and ligated kidneys, respectively, with no significant change during the 3‐ to 14‐day time course of UUO (Figure S5D). The collagen‐1 positive area also increased during UUO (Figure S6A,B). When choosing a threshold above the basal cellular (and extracellular) levels, on average only approx. 5%, 15% and 16% of all tagged REP reporter cells showed high levels of collagen‐1 after 3, 7 and 14 days, respectively, with no significant difference between the ligated and contralateral kidneys (Figure S6C). Of the small number of αSMA^+^/tdTomato^+^ double‐positive REP cells, on average 13%, 44% and 46% also expressed high levels of collagen‐1 after 3, 7 and 14 days, respectively, of UUO (Figure S6D), supporting the conclusion that this subset of αSMA^+^/tdTomato^+^ cells indeed transdifferentiated. However, the majority of tagged REP reporter cells did not increase the expression of the fibrotic markers αSMA and collagen‐1 during kidney disease, demonstrating that the rapid and severe initial loss of Epo mRNA expression is not caused by myofibroblast transdifferentiation of REP cells.

### 2.7 FG‐4592 efficiently reactivates Epo expression in formerly active REP cells of the diseased kidney

**FIGURE 7:**
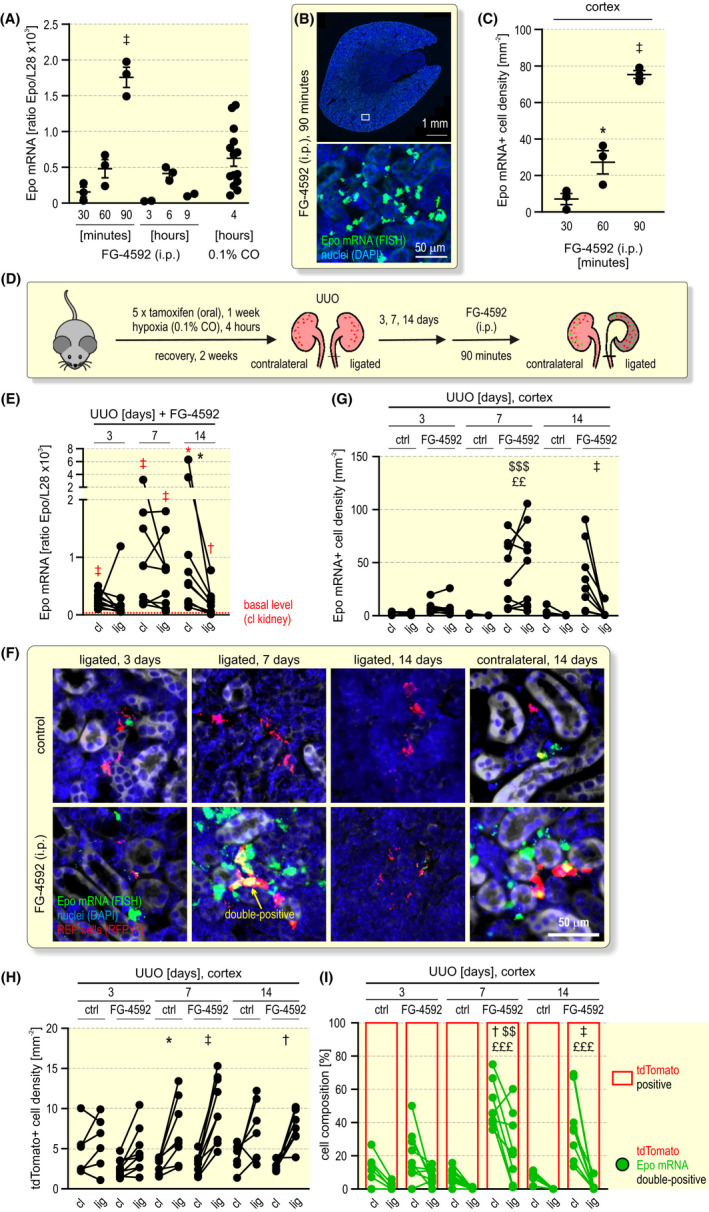
FG‐4592 (roxadustat) treatment of mice with unilateral ureteral obstruction. A, Kinetics of Epo induction after a single intraperitoneal (i.p.) injection of 50 mg kg^–1^ FG‐4592 into *Epo‐Cre^ERT2^#1*x*tdTomato* mice. Kidney Epo mRNA levels were determined by RT‐qPCR and are displayed relative to the ribosomal protein L28 mRNA levels. For comparison, Epo mRNA levels after 4 hours of 0.1% CO exposure were included. B, Detection of Epo mRNA positive REP cells by FISH (green) 90 minutes after FG‐4592 treatment. Nuclei were stained with DAPI (blue). C, Quantification of Epo mRNA^+^ REP cells at the indicated time points after FG‐4592 treatment. A, C, One‐way ANOVA followed by Dunnett’s post‐hoc correction was used to statistically evaluate Epo induction versus the earliest (30 minutes) FG‐4592 treatment (average ± SEM; *P < .05, ^‡^ *P* < .001). D, Schematic illustrating the conditional tagging of REP reporter cells followed by unilateral ureteral obstruction (UUO) and a single i.p. injection of FG‐4592. Kidneys were excised 90 minutes later. E, Epo mRNA levels of contralateral (cl) and ligated (lig) kidneys, quantified as in (A); the dashed red line indicates the average basal Epo mRNA levels of the contralateral kidney of the non‐FG‐4592‐treated mice shown in Figure 5B. Student’s *t* tests were used to statistically evaluate Epo induction versus the basal Epo levels (*P < .05, ^†^ *P* < .01, ^‡^ *P* < .001; red symbols). Repeated measures two‐way ANOVA followed by Bonferroni’s post‐hoc correction was used to statistically evaluate Epo changes in the ligated versus contralateral kidneys (*P < .05). F, Detection of Epo mRNA^+^ (FISH, green) and tdTomato^+^ (anti‐RFP, red) REP cells. Quantification of Epo mRNA^+^ (G) and tdTomato^+^ (H) REP cell densities of untreated (ctrl) mice or following treatment with FG‐4592. Because of the loss of medullary tissue during UUO, this analysis focused on the cortex. The values of 120 to 800 cells per timepoint are shown. I, tdTomato positive (red) and tdTomato/Epo mRNA double‐positive (green) cell composition. G–I, Repeated measures three‐way ANOVA followed by Bonferroni’s post‐hoc correction was used to statistically evaluate Epo changes in the ligated versus contralateral kidneys (*P < .05, ^†^ *P* < .01, ^‡^ *P* < .001), in the FG‐4592 treated ligated versus untreated ligated kidneys (^$$^ *P* < .01, ^$$$^ *P* < .001), and in the FG‐4592 treated contralateral versus untreated contralateral kidneys (^££^ *P* < .01, ^£££^ *P* < .001)

FG‐4592 is in clinical use for the treatment of renal anaemia, and the (diseased) kidney has been implicated in the production of circulating Epo induced by pharmaceutical HIF stabilization.^28^ However, the actual renal cellular source of Epo in FG‐4592 treated patients is unclear, that is, whether previously active REP cells are still capable of producing Epo in response to pharmacological stimulation and/or whether an independent set of cells from the dormant “off” REP cell pool is recruited. To answer this question, we first investigated the Epo induction kinetics following a single i.p. injection of FG‐4592. We observed a peak of renal Epo mRNA induction as soon as after 90 minutes, while no or much lower induction of Epo mRNA could be detected after 3 to 9 hours (Figure 7A). Intriguingly, this early transient Epo mRNA peak reached even higher levels than the 4 hours 0.1% CO exposure. Strong Epo mRNA production by FG‐4592 was confirmed by FISH (Figure 7B). Quantification revealed a ~10‐fold induction of Epo mRNA^+^ REP cells from 30 to 90 minutes of FG‐4592 treatment (Figure 7C).

Based on these data, mice with tagged REP reporter cells were treated 3, 7 or 14 days after the onset of UUO for 90 minutes with FG‐4592 (Figure 7D). Compared to the basal Epo mRNA levels of untreated mice, Epo expression increased in almost all kidneys analysed. Importantly, Epo mRNA induction in the diseased (ligated) kidney was largely similar to the healthy (contralateral) kidney after 7 days of UUO (Figure 7E). After 14 days of UUO, excessive tissue damage occurred, as assessed by DNA fragmentation and tubular disintegration (Figure S7A). Cleaved caspase 3 accumulation revealed strong apoptosis in some but not all ligated kidneys, and an inverse correlation was observed between the extent of tissue apoptosis and Epo responsiveness to FG‐4592 (Figure S7B‐D). Consistently, after 14 days of UUO FG‐4592‐dependent Epo induction was less pronounced in the diseased than in the healthy kidney but was still significantly above the basal Epo mRNA levels (Figure 7E). These results were confirmed by Epo mRNA FISH on the single REP cell level (Figure 7F). Quantification corroborated the comparable induction of Epo mRNA positive REP cells by FG‐4592 in the healthy and diseased kidneys after 7 days of UUO, that is, before tissue damage became excessive (Figure 7G).

Quantification of the tdTomato^+^ cells consistently demonstrated the proliferation of tagged REP cells also in this series of UUO experiments (Figure 7H). After 7 days of UUO, FG‐4592 recruited on average ~49% and ~28% of the tagged REP reporter cells in the healthy and diseased kidneys, respectively (Figure 7I). Compared with the CO/hypoxia recruitment of ~60% tagged REP cells in healthy kidneys (see Figure 3H above), FG‐4592 remarkably recruited half as many tagged REP reporter cells in the diseased kidney after 7 days of UUO.

Using sensitive FISH to detect αSMA mRNA, we first independently confirmed the low portion (on average ~17%) of tdTomato^+^ REP reporter cells undergoing myofibroblast differentiation (Figure S8A,B), ruling out a possible insensitive αSMA protein detection. We then used αSMA/Epo double‐FISH to analyse the contribution of these two REP cell subsets to FG‐4592‐induced Epo mRNA. However, after 7 days of kidney ligation the fraction of αSMA^+^ REP cells that responded to FG‐4592 was similar to the overall fraction of αSMA positive REP cells (Figure S8C,D), suggesting that also the αSMA^+^ subset of recently active REP cells contributes to FG‐4592‐induced Epo production in CKD.

## 3 DISCUSSION

Due to the rapid and transient nature of hypoxic Epo mRNA induction in the kidney, tagging of REP cells in transgenically altered reporter mouse models is required to trace the fate of these cells. Typically, severe anaemia by ~50% phlebotomy^15^, ^18^, ^27^, ^29^, ^30^, ^31^ or “inherited super‐anaemic mice”^15^, ^27^ have been used to study REP cells, limiting the physiological relevance of these mouse models. In contrast, we used a brief CO hypoxaemia stimulus that blocks for a short period ~50% of the blood oxygen transport capacity but with much faster recovery and less systemic side effects than anaemia, allowing for the time‐dependent analysis of these cells in a more physiological context shortly after tagging. Like previously optimized for Cre^ERT2^ activation,^32^ tamoxifen was applied in 5 daily doses. Following the rapid reversal of the hypoxic conditions, tamoxifen‐mediated Cre^ERT2^ activation is known to persist for several days,^33^ contributing to the observed increase in tdTomato^+^ REP reporter cells during the first week, but thereafter the number and localization of the labelled REP cells remained stable during the entire observation period. There was no sign of transdifferentiation, and a repeated hypoxic stimulus stochastically recruited a REP cell population that partially overlapped with these previously active REP reporter cells, no matter how long ago the initial hypoxic stimulus was applied.

An important finding of our study, with implications for the treatment of renal anaemia, was the loss of Epo preceding a rather small increase in αSMA expression in REP cells during progressive renal tissue remodelling. Phenotypic changes of Epo‐producing cells have previously been observed, which led to the suggestion that loss of Epo expression and development of renal anaemia during CKD may at least partially be a consequence of transdifferentiation of Epo‐producing cells into myofibroblasts.^34^, ^35^ Indeed, in a UUO mouse model with a reporter gene knock‐in into the *Epo* locus, 50% and 80% of REP cells have been reported to become αSMA positive as soon as 2 and 3 days, respectively, after ureter ligation.^27^ In another REP cell tagging model, 80% tdTomato^+^ REP cells were αSMA positive after 14 days of UUO.^27^ However, these experiments have been performed in a background of genetically generated congenital anaemia, leading to the premature death of the mice.^15^, ^36^ While severe anaemia allowed for the tagging of a (unphysiological) large number of REP cells, it limited the relevance of these mouse models for the investigation of renal anaemia. In contrast, in our mouse model we tagged only the currently most active REP cells which then served as a genuine REP cell reporter population. Our data confirmed the previously reported induced proliferation of interstitial cells and increase in fibrotic markers during UUO.^37^ However, we conclude that myofibroblast differentiation is not the primary cause for the initial failure of Epo expression in the diseased kidney, suggesting that REP cells remained in a potentially Epo‐expressing dormant state. This suggestion was supported by the finding that FG‐4592/roxadustat could restore Epo mRNA transcription in the very same cells.

Interestingly, in healthy mice the spatial distribution of Epo mRNA positive REP cells following pharmacological induction by FG‐4592 appeared to be similar to the physiological distribution in the corticomedullary border region following hypoxic exposure. Thus, either an unknown mechanism governs the preferred spatial localization of recruitable REP cells, or cooperation between FG‐4592 and local tissue hypoxia is necessary for sufficient HIF stabilisation to induce Epo transcription. FG‐4592 maintains a Hb value at the lower edge of the normal range.^17^ Indeed, with the here used FG‐4592 dose only about a quarter of tdTomato^+^ REP cells were counted compared with 8% O_2_ or 0.1% CO inspiration, supporting the assumption of a cooperative activity between FG‐4592 and tissue hypoxia. Thus, the unique spatial localization of REP cells within the renal oxygen gradient may contribute to the apparent Epo‐inducing specificity of this pan‐HIF‐stabilizing drug.^38^

In conclusion, our results suggest that REP cell functionality persists during long‐term phases of inactivity in normoxia as well as during myofibrotic tissue remodelling. The findings that Epo expression is lost before myofibrotic markers are increased, and that pharmaceutical HIF stabilizers can efficiently rescue Epo expression in dormant and resident, previously active REP cells of diseased kidneys, are consistent with a suspected hyperoxic microenvironment rather than epigenetically fixed transdifferentiation of REP cells.

## 4 MATERIALS AND METHODS

### 4.1 Mice

The generation of *Epo‐Cre^ERT2^#1* (B6D2;C57BL6N‐Tg (EPO::Cre)_n_Rhw) mice and the crossing with Ai14 reporter (B6.Cg‐Gt(ROSA)26Sor<tm14(CAG‐tdTomato)Hze>/J) mice as well as their exposure to 8% O_2_ or 0.1% CO has previously been described.^7^ Tamoxifen (T5648; Sigma‐Aldrich, St Louis, MO, USA) was dissolved in 10% ethanol and 90% sunflower or corn oil (50 mg mL^–1^) by ultrasonication (Bioruptor Plus with built‐in cooling system; Diagenode, Seraing, Belgium) using 5 to 10 cycles (30 seconds on/off) until in solution. Tamoxifen (200 mg kg^–1^) was applied daily for 5 days by gavage. Where indicated, 50 mg kg^–1^ FG‐4592/roxadustat (Selleckchem, Houston, Texas, USA), dissolved at 20 mg mL^–1^ in 0.5 M Tris‐HCl pH 9.0, was co‐applied with tamoxifen by gavage daily for 5 days to tag REP cells, or once intraperitoneally injected to analyse the acute Epo mRNA regulation. For UUO, mice were anaesthetized by isofluorane inhalation under buprenorphine analgesia. Bladder and ureter were exposed through an abdominal incision and the left ureter was ligated with two surgical knots of non‐absorbable silk suture. For sham operations, the ureter was exposed without ligation. Mice were killed by cervical dislocation. The details of the mouse trials (numbers, sex, survivors) are listed in the CONSORT flow diagrams (www.consort‐statement.org) provided in Figure S9. All animal experiments were approved by the veterinary office of the canton Zurich (license numbers ZH233/2015, ZH200/2016 and ZH085/2019).

### 4.2 Blood analyses

Blood was collected from the submandibular vein. Haematocrit was measured using a microhaematocrit centrifuge in heparin‐coated capillary tubes. Haemoglobin concentration and CO saturation was assessed with a whole‐blood oximeter (Avoximeter 1000; Instrumentation Laboratory, Bedford, MA, USA). Circulating Epo protein levels were quantified by ELISA according to the manufacturer’s instructions (MEP00B; R&D Systems, Minneapolis, MN, USA).

### 4.3 mRNA quantification

Transcript levels were quantified by reverse transcription (RT) real‐time quantitative (q) PCR and normalized to the mRNA levels of the housekeeping ribosomal proteins L28 or S12 as described before.^7^, ^39^ Primers (Microsynth, Balgach, Switzerland) are listed in Table S1.

### 4.4 Immunofluorescence analyses

Excised kidneys were cut in half and fixed with 4% paraformaldehyde overnight at 4°C. For cryosections, kidneys were dehydrated with 30% sucrose, embedded in optimum cutting temperature (OCT) compound (Tissue‐Tek; Sakura Finetek, Alphen aan den Rijn, The Netherlands) and cut in 12 µm sections using a cryotome. Cryosections were blocked and permeabilized with 5% normal goat serum/0.3% Triton X‐100 in PBS and incubated with primary antibodies overnight at 4°C. After washing 3 times in PBS the sections were incubated with secondary antibodies for 1 hour at room temperature. The following antibodies were used: rabbit polyclonal anti‐Ki67 (ab15580; Abcam, Cambridge, UK); rabbit polyclonal anti‐αSMA (ab5694; Abcam); mouse monoclonal anti‐collagen 1 (MA1‐26771; Invitrogen, Carlsbad, CA, USA); rabbit monoclonal anti‐vimentin (ab92547; Abcam); rabbit monoclonal anti‐cleaved caspase‐3 (5A1E; Cell Signaling; Danvers, MA, USA); secondary goat antibodies coupled to Alexa488 or Alexa647 (Thermo Fisher Scientific, Waltham, MA, USA). Details of the immunofluorescence procedures are provided in Table S2. DNA fragmentation was detected using a Click‐iT TUNEL Alexa Fluor imaging assay according to the manufacturer’s instructions (Invitrogen). Nuclei were counterstained with 0.5 µg mL^–1^ 4′,6‐diamidino‐2‐phenylindole (DAPI; Sigma‐Aldrich). The sections were mounted in Mowiol (Sigma‐Aldrich) and fluorescent signals were recorded using a slide scanner (Axio Scan.Z1; Zeiss Microscopy, Feldbach, Switzerland).

### 4.5 mRNA fluorescence in situ hybridization (FISH)

Cryosections were pre‐treated according to the manufacturer’s recommendations (RNAscope Multiplex Fluorescent v2 Assay; Advanced Cell Diagnostics, Hayward, CA, USA). The Epo probe (Cat No. 315501) consisted of 12 double Z probe pairs, targeting the region between 39 and 685 of mouse Epo mRNA. The αSMA probe (Cat No. 319531) consisted of 20 double Z probe pairs, targeting the region between 41 and 1749 of mouse Acta2 mRNA. The probes were hybridized for 2 hours at 40°C in a HybEZ oven (Advanced Cell Diagnostics), followed by signal amplification and signal detection using Opal 570 or Opal 650 fluorescent reagents (Akoya Biosciences, Marlborough, MA, USA). Because the endogenous tdTomato fluorescence was destroyed by the FISH procedure, for co‐detection of tdTomato protein sections were blocked with 10% normal goat serum/1% BSA in TBS for 1 hour at room temperature and incubated with rabbit polyclonal anti‐RFP (600‐401‐379; Rockland, Limerick, PA, USA) overnight at 4°C. After washing 5 times in 0.05% Tween‐20 in TBS, the sections were incubated with goat anti‐rabbit secondary antibody coupled to Alexa568 (Thermo Fisher Scientific) for 1 hour at room temperature. Sections were washed, counterstained with DAPI, mounted using ProLong Gold antifade mountant (Thermo Fisher Scientific) and fluorescent signals were recorded using the Axio Scan.Z1 slide scanner.

### 4.6 Image analyses

Whole kidney slice images were converted to 8‐bit grayscale images and whole slice analysis was performed with MATLAB R2018a (The Mathworks, Natick, MA, USA). Endogenous tdTomato signal was binarized with Otsu’s method and background subtracted. Fluorescent signals with a minimum diameter of 10 µm were counted as tdTomato^+^ cells. Kidney area was determined using DAPI fluorescence. The signal was smoothened with a Gaussian filter followed by morphological close operation to merge the complete region. Kidney segments were distinguished by green autofluorescence images, allowing region of interest selection based on anatomy and morphology. For total cell estimation per kidney, the kidney volume was calculated with the ellipsoid equation. The length was measured in centrically sections as maximum longitudinal diameter. Depth and width were measured in centrically transverse sections. Cell quantifications from 2D sections were extrapolated to the estimated kidney volume. For IF image analysis, gray scale thresholds were arbitrarily selected and maintained throughout the experiment. After binarization, tissue positive area and co‐localization with tdTomato^+^ cells was calculated. A minimal overlap of 10 pixels was defined as double‐positive. For FISH image analysis, every fluorescent dot was manually annotated using ZEN 3.1 (Zeiss Microscopy). Dots forming clusters in maximal proximities of 5 µm were considered the same cell origin. RFP‐labelled tdTomato^+^ cells were counted manually. Image analyses were performed in a blinded fashion.

### 4.7 Statistical analyses

Statistical analyses were performed using Prism 9 (GraphPad Software, San Diego, CA, USA). Kolmogorov‐Smirnov (n > 5) or Kruskal‐Wallis (n < 5) tests and QQ plots were used as a pre‐hoc analyses to assure normal distribution of the data. Student’s *t* tests were used to statistically evaluate pairwise differences. Time courses were assessed by one‐way analysis of variance (ANOVA) and UUO experiments were assessed by repeated measures two‐ or three‐way ANOVA, followed by the appropriate post‐hoc tests as indicated in the figure legends. *P*‐values < .05 were considered as significant.

### 4.8 Data sharing information

All the material submitted is conformed to good publishing practice in physiology.^40^ Underlying raw data can be obtained from RHW (roland.wenger@access.uzh.ch) and are available via https://dataverse.harvard.edu/ using the accession code https://doi.org/10.7910/DVN/2RV9EV.

## CONFLICT OF INTEREST

The authors declare that no conflict of interest exists.

## AUTHOR CONTRIBUTIONS

SLD, KAN, SP, XD and TK performed experiments in mice. SLD, KAN and RH analysed the tissues. SLD and RHW analysed the data. PS and CCS provided technical and intellectual input. KAN and RHW designed the research. RHW wrote the article.

## Supporting information

Supplementary Material

## Data Availability

Underlying raw data can be obtained from RHW (roland.wenger@access.uzh.ch) and are available via https://dataverse.harvard.edu/ using the accession code https://doi.org/10.7910/DVN/2RV9EV.
