# Supplementary material for: Fate‐mapping of erythropoietin‐producing cells in mouse models of hypoxaemia and renal tissue remodelling reveals repeated recruitment and persistent functionality
Source: Acta Physiol (Oxf). 2022 Jan 16;234(3):e13768. doi: 10.1111/apha.13768 (PMC9286872; doi:10.1111/apha.13768)
Supplement: Supplementary file 1 — Supplementary Material [file APHA-234-0-s001.pdf]

SUPPORTING INFORMATION

Fate-mapping of erythropoietin-producing cells in mouse models of hypoxaemia and renal tissue remodelling reveals repeated recruitment and persistent functionality.

Sophie L. Dahl, Svende Pfundstein, Rico Hunkeler, Xingtong Dong, Thomas Knöpfel, Patrick Spielmann, Carsten C. Scholz, Karen A. Nolan, Roland H. Wenger

Table of Contents

Table S1. Primers used for PCR.
Table S2. Reagents and procedures used for immunoflourescence.
Figure S1. Cellular Epo mRNA frequency distribution.
Figure S2. Equal distribution of REP reporter cells within full kidney.
Figure S3. Epo and Cre^{ERT2} mRNA decline following reoxygenation.
Figure S4. Expression of the Ki67 proliferation marker following REP cell tagging.
Figure S5. Expression of the mesenchymal marker vimentin in the diseased kidney.
Figure S6. Expression of the extracellular matrix protein collagen-1 in the diseased kidney.
Figure S7. DNA fragmentation and apoptosis in the diseased kidney.
Figure S8. FG-4592-dependent Epo mRNA induction in αSMA mRNA positive and negative REP cells.
Figure S9. Flow diagrams of the mouse trials.

TABLE S1. Primers used for PCR

Epo (exons 3/4-4, 174 bp)
fwd 5'-aatggaggtggaagaacagg-3' rev 5'-acccgaagcagtgaagtga-3'

αSMA (exons 6/7-8, 331 bp)
fwd 5'-gactactgccgagcgtgag-3' rev 5'-gtcagcaatgcctgggtaca-3'

Vimentin (exons 1/2-4, 246 bp)
fwd 5'-ctgcgagagaaattgcaggag-3' rev 5'-agtgaggtcaggcttggaaa-3'

Cre^{ERT2} (cDNA, 203 bp)
fwd 5'-ataccggagatcatgcaagc-3' rev 5'-aaggccaggctgttcttctt-3'

L28 (exons 3-5, 196 bp)
fwd 5'-gcaaaggggtcgtggtagtt-3' rev 5'-ttctggcttcgaaggatggc-3'

S12 (exons 3-5/6, 235 bp)
fwd 5'-gaagctgccaaagccttaga-3' rev 5'-catagtccttaaccactacgcaac-3'

TABLE S2. Reagents and procedures used for immunoflourescence

Primary antibody	Reference	Company	Dilution	Procedure
αSMA	ab5694	Abcam	1:100	(1)/(2)
vimentin	ab92547	Abcam	1:1000	(1)
cleaved caspase 3	5A1E	Cell Signaling	1:300	(1)
collagen-1	MA1-26771	Invitrogen	1:500	(3)
Ki67	ab15580	Abcam	1:50	(4)
RFP	600-401-379	Rockland	1:200	(5)

Secondary antibody	Reference	Company	Dilution	Procedure
anti-rabbit Alexa Fluor 488	A-11008	Invitrogen	1:1000	(6)
anti-rabbit Alexa Fluor 568	A-11011	Invitrogen	1:1000	(7)
anti-rabbit Alexa Fluor 647	A-21244	Invitrogen	1:200/1:1000	(6)
anti-mouse Alexa Fluor 488	A-11001	Invitrogen	1:1000	(6)

Procedure

(1) blocking: 5% NGS/1% BSA
incubation: PBS/1% BSA/0.3% Triton X-100, 4°C, overnight
secondary antibody: anti-rabbit Alexa Fluor 647, 1:1000

(2) blocking: 5% NGS/1% BSA
incubation: PBS/1% BSA/0.3% Triton X-100, 4°C, overnight
secondary antibody: anti-rabbit Alexa Fluor 488, 1:1000

(3) blocking: 5% NGS/1% BSA
incubation: PBS/1% BSA/0.3% Triton X-100, 4°C, overnight
secondary antibody: anti-mouse Alexa Fluor 488, 1:1000

(4) blocking: 5% NGS/1% BSA
incubation: PBS/1% BSA/0.3% Triton X-100, 4°C overnight
secondary antibody: anti-rabbit Alexa Fluor 647, 1:200

(5) blocking: 10% NGS/1% BSA
incubation: TBS, 4°C, overnight
secondary antibody: anti-rabbit Alexa Fluor 568, 1:1000

(6) incubation: PBS/1% BSA/0.3% Triton X-100, room temperature, 1 hour
dilution: see (1)-(4)

(7) incubation: TBS, room temperature, 1 hour
dilution: see (5)

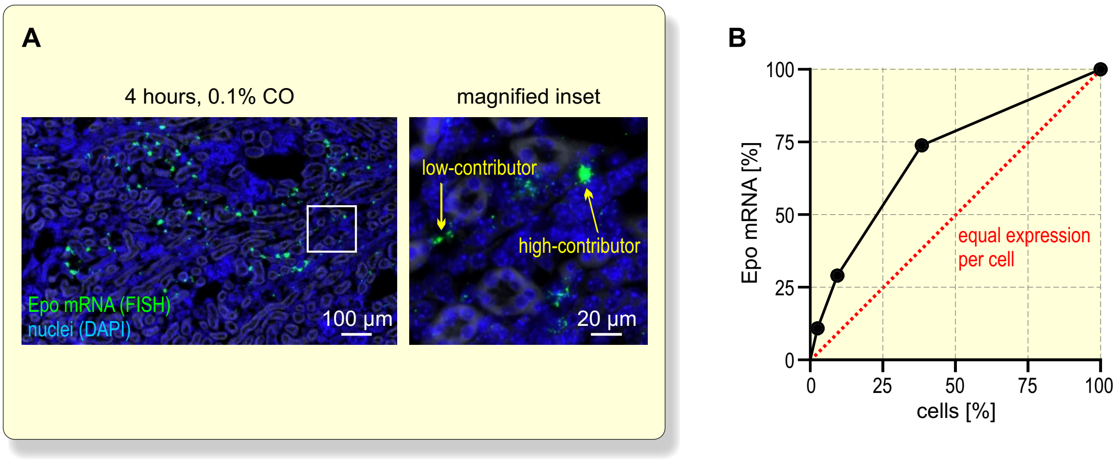

FIGURE S1 Heterogeneity of cellular Epo mRNA expression. (A) Chosen region of interest for analysis. Detection of Epo mRNA positive REP cells by FISH (green) and nuclei by DAPI staining (blue). Exemplary cells with a high amount of Epo mRNA (high-contributor) and low amount of Epo mRNA (low-contributor) are depicted. (B) Cumulative frequency distribution of cellular Epo mRNA. A total of 117 cells were analyzed. The dashed red line indicates equal cellular expression.

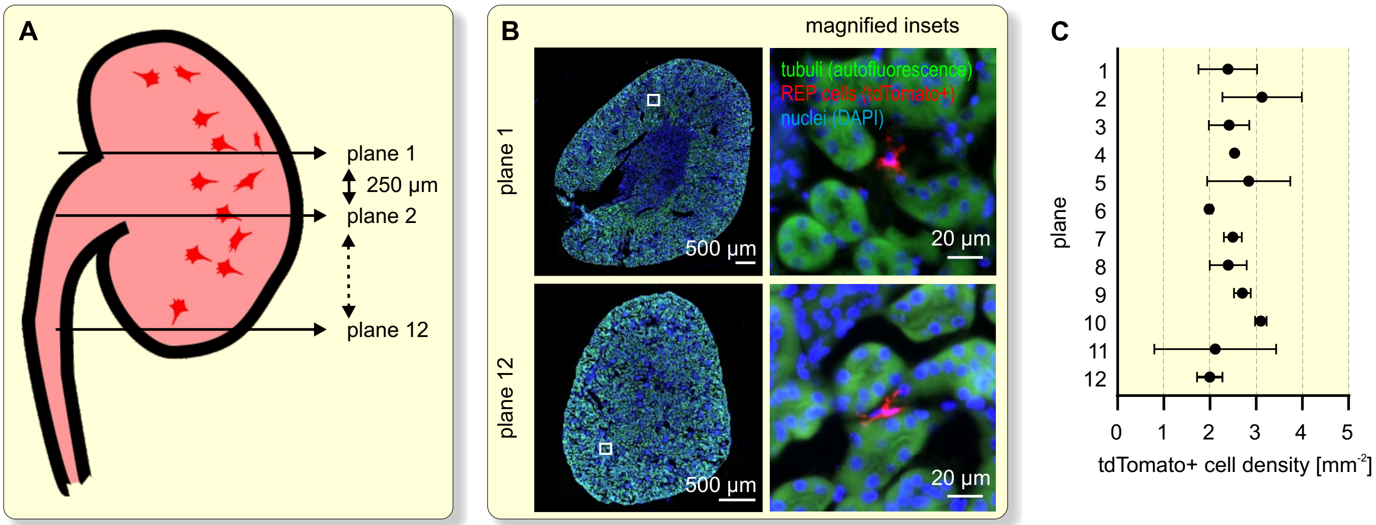

FIGURE S2 Equal distribution of REP cell density throughout the kidney. (A) Schematic illustrating the distribution of REP reporter cells: 32 weeks after the conditional tagging in *Epo-Cre^{ERT2}#1xtdTomato* mice, the kidney was cut at 12 different planes with a distance of 250 µm between each plane as indicated. (B) Detection of tdTomato^{+} REP reporter cells (red) in planes 1 and 12. Tubuli were visualized by their autofluorescence (green) and nuclei were stained with DAPI (blue). (C) Quantification of tdTomato^{+} REP cell density. Each data point represents the average value of 2 slices of 12 µm derived from one kidney at planes 1-12. Shown is the average per group ± SD.

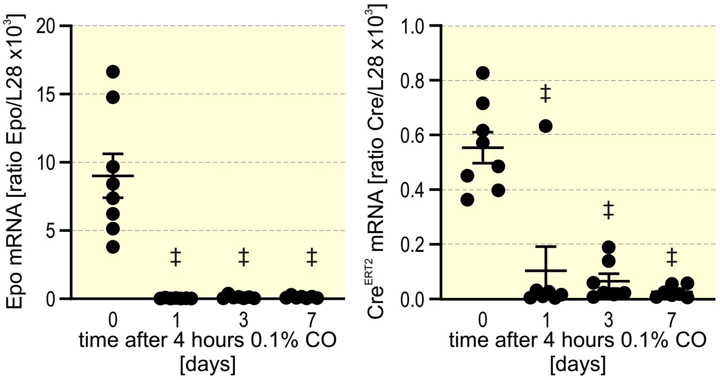

FIGURE S3 Epo and Cre^{ERT2} mRNA decline following reoxygenation. After exposure to 0.1% CO for 4 hours, mice were allowed to recover for the indicated time periods. Kidney Epo and Cre^{ERT2} mRNA levels were determined by RT-qPCR and are displayed relative to the ribosomal protein L28 mRNA levels. One-way ANOVA followed by Dunnett's post-hoc correction was used to statistically evaluate mRNA changes vs. the 0 days group ($^{‡}p<0.001$).

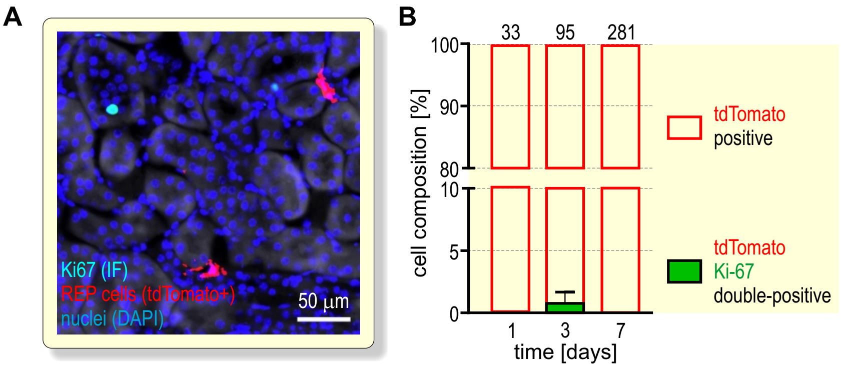

FIGURE S4 Expression of the proliferation marker Ki67 following REP cell tagging. (A) Example of Ki67 immunofluorescence (IF; cyan) combined with REP cell tagging in *Epo-Cre^{ERT2}#1xtdTomato* mice (red) and nuclear staining by DAPI (blue). (B) Quantification of tdTomato/Ki67 double-positive cells following the indicated days since tagging. For each timepoint 5 mice were combined. The number of analyzed tdTomato^{+} REP cells per timepoint is indicated.

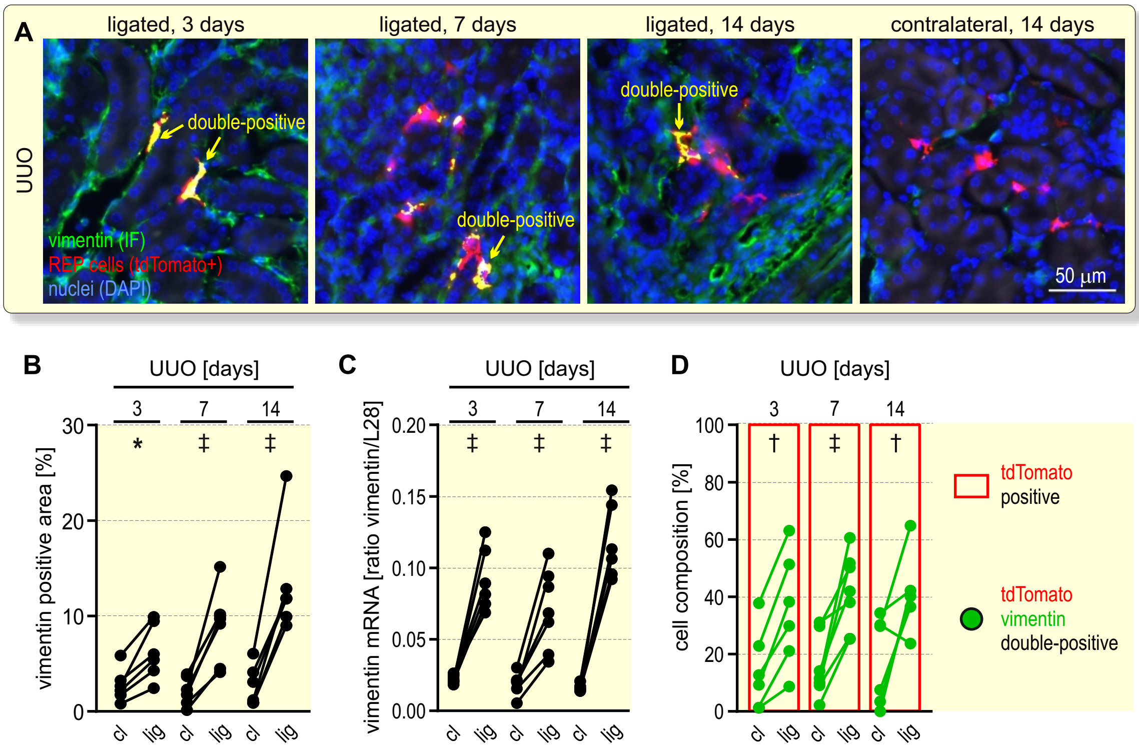

FIGURE S5 Expression of the mesenchymal marker vimentin in the diseased kidney. (A) Example of vimentin immunofluorescence (IF; green) combined with REP cell tagging in *Epo-Cre^{ERT2}#1xtdTomato* mice (red) and nuclear staining by DAPI (blue) in the contralateral and ligated kidneys 14 days after unilateral ureteral obstruction (UUO). (B) Automated quantification of the vimentin^{+} area in the contralateral (cl) and ligated (lig) kidneys. (C) Kidney vimentin mRNA levels were determined by RT-qPCR and are displayed relative to the ribosomal protein L28 mRNA levels. (D) tdTomato positive (red) and tdTomato/vimentin double-positive (green) cell composition. Labelled cells were automatically counted and the values of 850 to 1100 cells per timepoint are shown. (B-D) Repeated measures two-way ANOVA followed by Bonferroni's post-hoc correction was used to statistically evaluate vimentin changes in the ligated vs. contralateral kidneys (*$p<0.05$, $^{\dagger}p<0.01$, $^{\ddagger}p<0.001$).

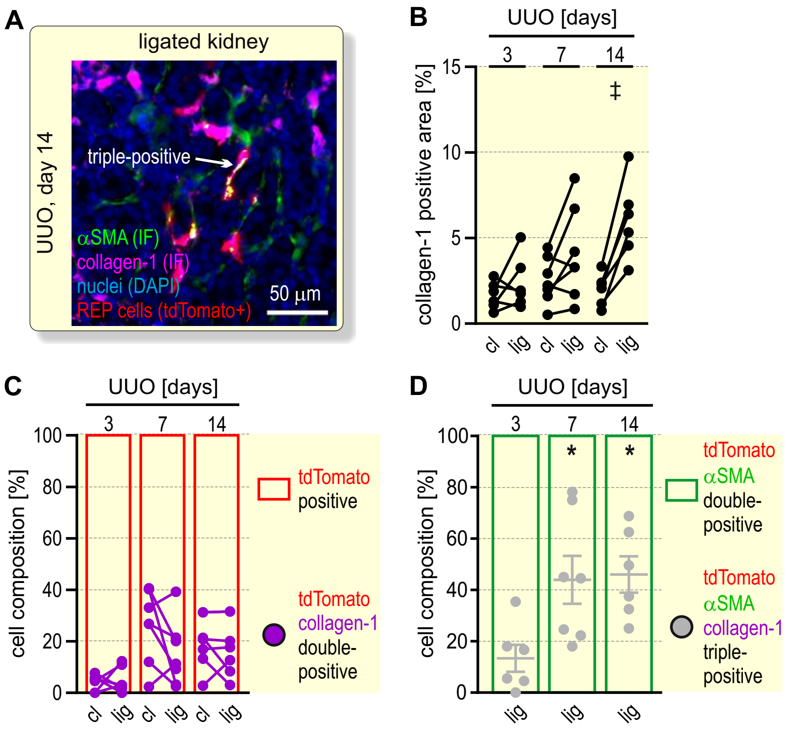

FIGURE S6 Expression of the extracellular matrix protein collagen-1 in the diseased kidney. (A) Example of cellular collagen-1 immunofluorescence (IF; magenta) combined with REP cell tagging in *Epo-Cre^{ERT2}#1xtdTomato* mice (red), αSMA IF (green), and nuclear staining by DAPI (blue) in a ligated kidney 14 days after unilateral ureteral obstruction (UUO). tdTomato/αSMA double-positive and tdTomato/αSMA/collagen-1 triple-positive pixels are depicted in yellow and white, respectively. To distinguish enhanced cellular collagen-1 expression during UUO from the basal expression levels in REP cells, a detection threshold above the extracellular collagen-1 levels was chosen. (B) Automated quantification of the collagen-1^{+} area in the contralateral (cl) and ligated (lig) kidneys. (C) tdTomato positive (red) and tdTomato/collagen-1 double-positive (magenta) cell composition. Labelled cells were automatically counted and the values of at least 800 cells per timepoint are shown. (B, C) Repeated measures two-way ANOVA followed by Bonferroni's post-hoc correction was used to statistically evaluate collagen-1 changes in the ligated vs. contralateral kidneys ($^{‡}p<0.001$). (D) tdTomato/αSMA double-positive (green) and tdTomato/αSMA/collagen-1 triple-positive (white) cell composition. Labelled cells were automatically counted and 16, 176 and 69 triple-positive cells were obtained after 3, 7 and 14 days of UUO, respectively. One-way ANOVA followed by Dunnett's post-hoc correction was used to statistically evaluate cell composition vs. the 3 days group ($^{*}p<0.05$).

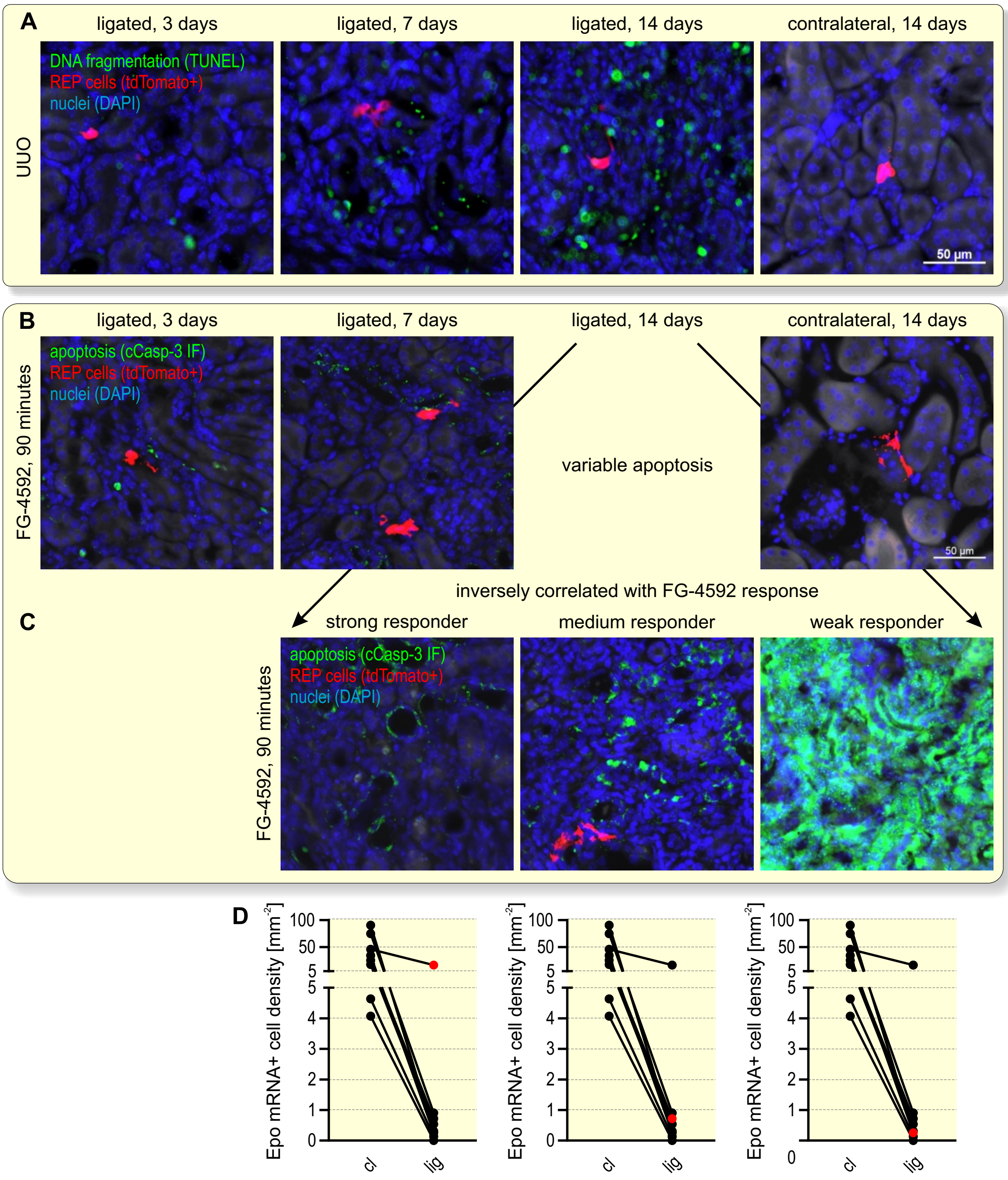

FIGURE S7 DNA fragmentation and apoptosis in the diseased kidney. (A) Examples of terminal deoxynucleotidyl transferase dUTP nick end labeling (TUNEL, green) combined with REP cell tagging in *Epo-Cre^{ERT2}#1xtdTomato* mice (red) and nuclear staining by DAPI (blue) in ligated kidneys 3, 7 and 14 days after unilateral ureteral obstruction (UUO). An example of a contralateral kidney after 14 days of UUO is shown on the right. (B) Examples of cleaved caspase-3 (cCasp-3) immunofluorescence (IF; green) combined with REP cell tagging (red) and nuclear staining by DAPI (blue) in ligated kidneys 3, 7 and 14 days after UUO and 90 minutes after FG-4592 i.p. injection, including an example of a contralateral kidney. (C) For ligated kidneys 14 days after UUO, example pictures of kidneys with variable FG-4592 response are shown. (D) Duplication of the density of Epo mRNA^{+} cells shown in **Figure 7G** (note the different y-axis). The red dots correspond to the sample kidneys shown above each graph.

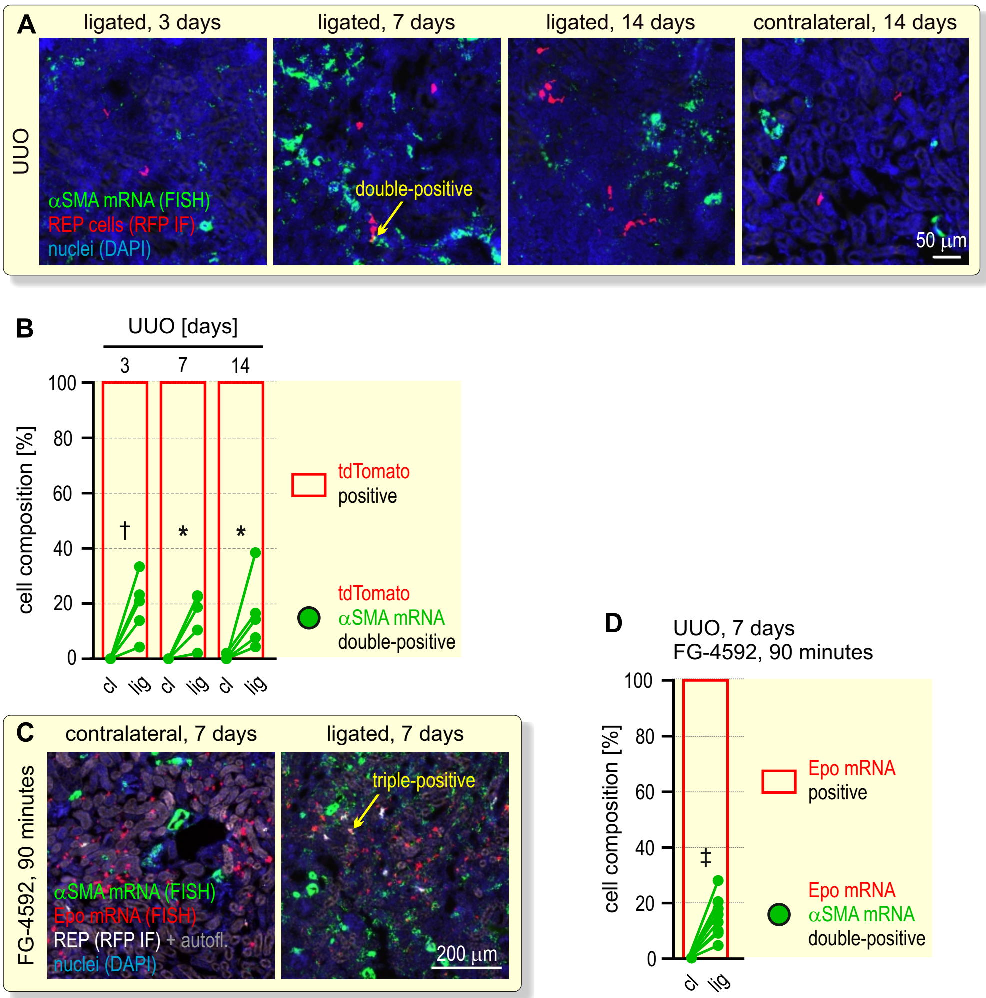

FIGURE S8 FG-4592-dependent Epo mRNA induction in αSMA mRNA positive and negative REP cells. (A) Examples of αSMA mRNA fluorescence *in situ* hybridization (FISH; green) combined with immunofluorescence detection of tdTomato in tagged REP cells by an anti-red fluorescent protein (RFP) antibody (red) and nuclear staining by DAPI (blue), 3, 7 and 14 days after unilateral ureteral obstruction (UUO). An example of a contralateral kidney after 14 days of UUO is shown on the right. (B) tdTomato positive (red) and tdTomato/αSMA mRNA double-positive (green) cell composition of the contralateral (cl) and ligated (lig) kidneys. Labelled cells were automatically counted and the values of at least 195 cells per timepoint are shown. Repeated measures two-way ANOVA followed by Bonferroni's post-hoc correction was used to statistically evaluate cell composition in the ligated vs. contralateral kidneys (*$p<0.05$; $^{\dagger}p<0.01$). (C) Examples of αSMA mRNA FISH (green) combined with Epo mRNA FISH (red), RFP immunofluorescence detection of tdTomato^{+} tagged REP cells (white), tubular autofluorescence (gray) and nuclear staining by DAPI (blue), 7 days after UUO and 90 minutes after FG-4592 i.p. injection. (D) Epo mRNA positive (red) and Epo/αSMA mRNA double-positive (green) cell composition of the contralateral (cl) and ligated (lig) kidneys. Labelled cells were automatically counted and the values of at least 220 cells per timepoint are shown. Paired Student's *t*-test was used to statistically evaluate αSMA changes in the ligated vs. contralateral kidneys ($^{\ddagger}p<0.001$).

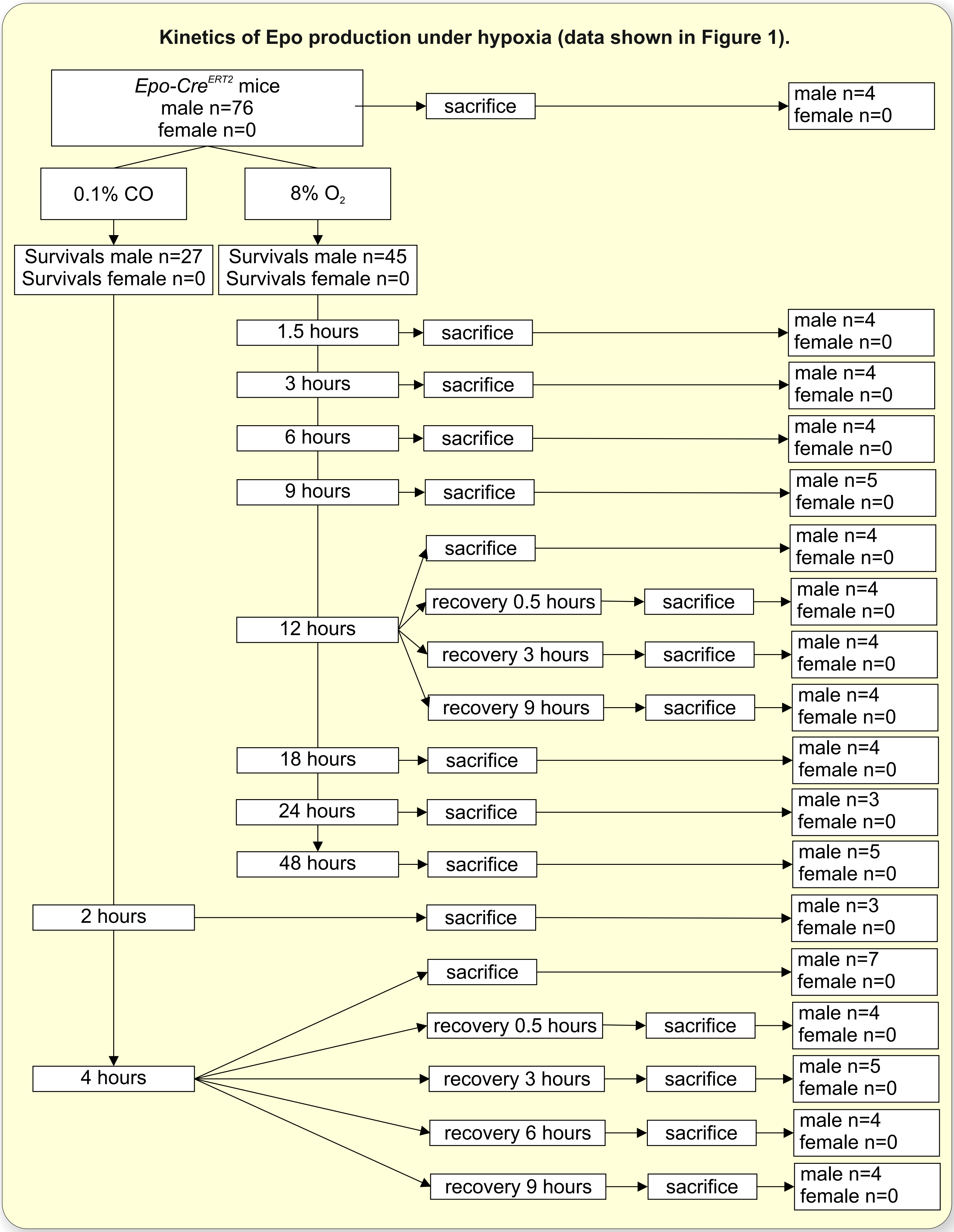

FIGURE S9 Flow diagrams of the mouse trials.

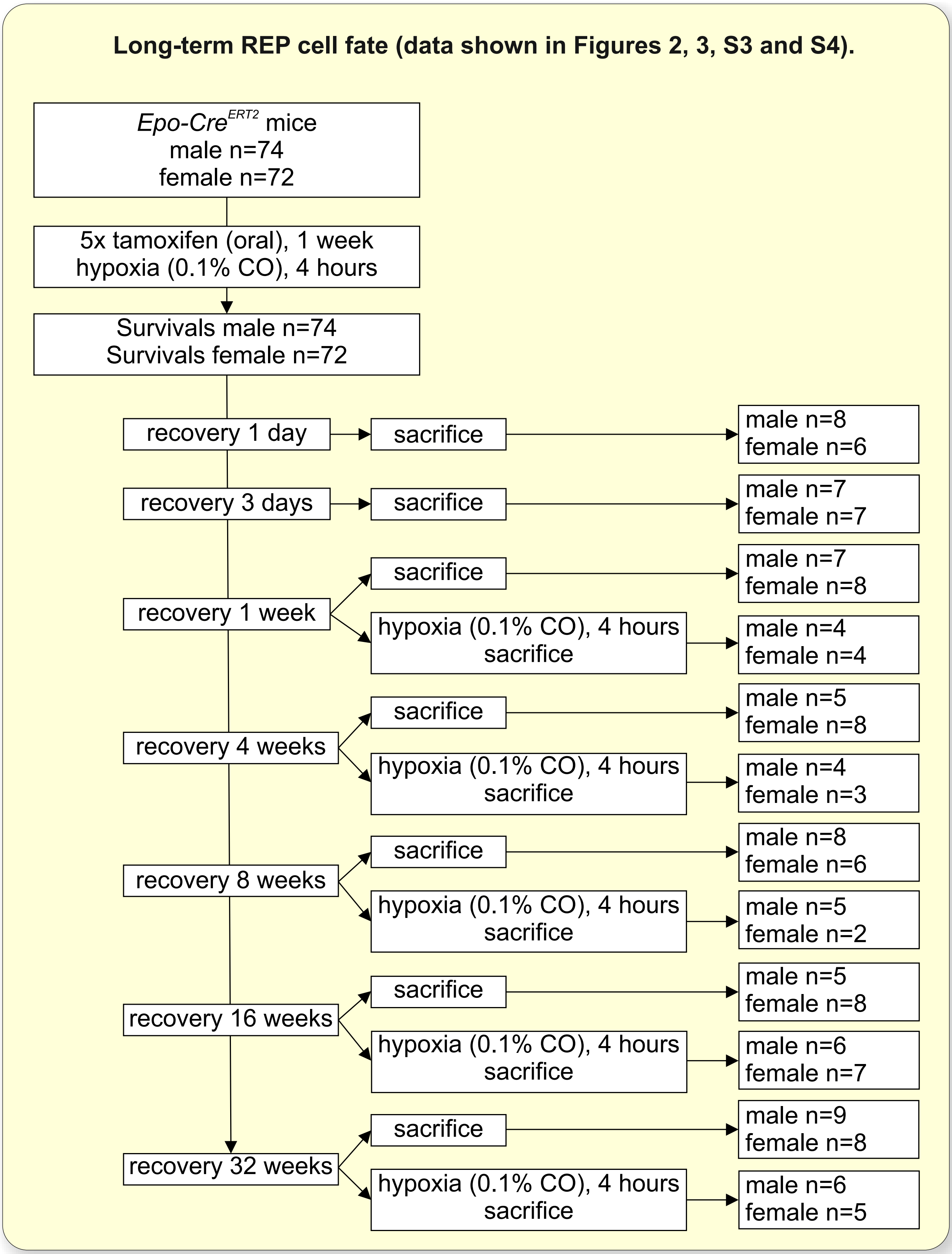

FIGURE S9 Flow diagrams of the mouse trials.

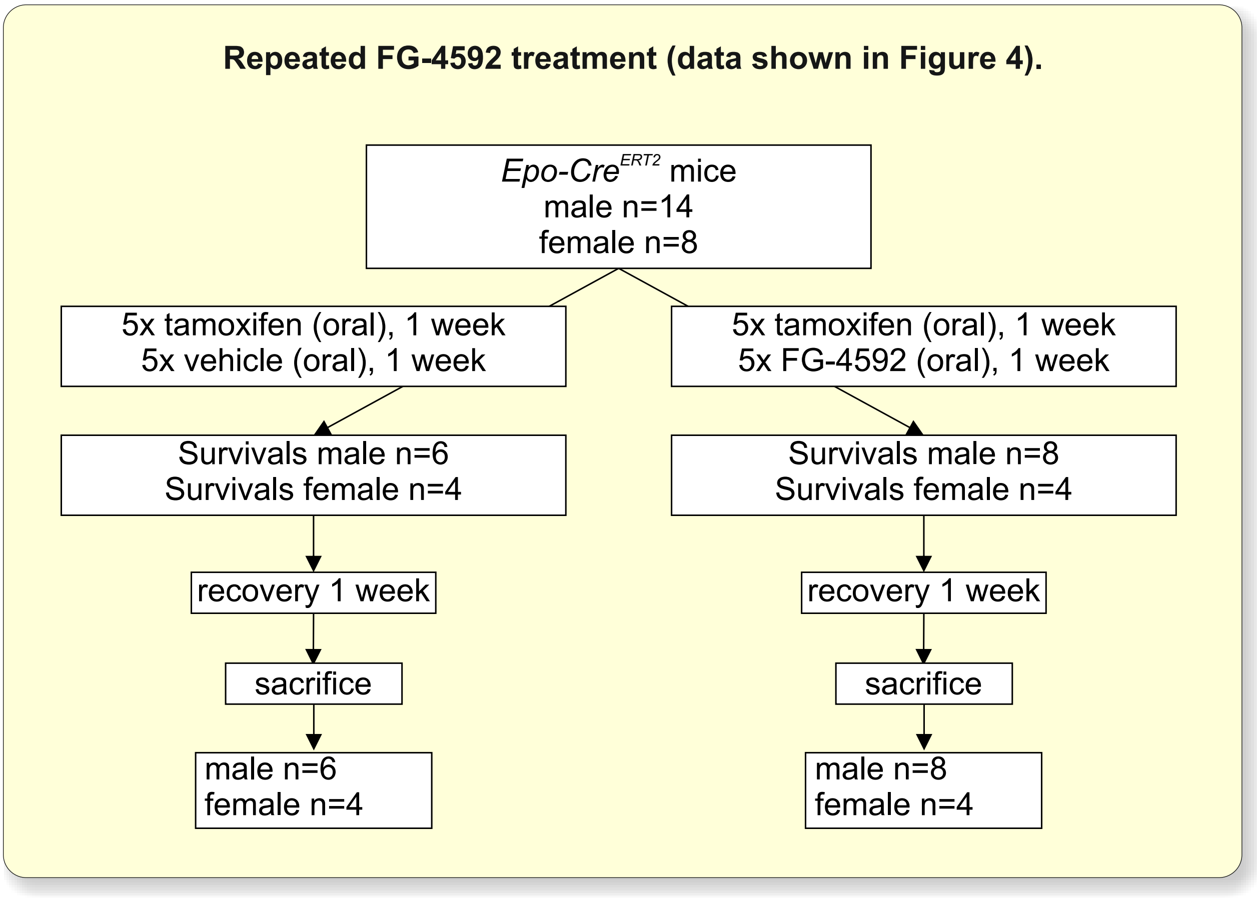

FIGURE S9 Flow diagrams of the mouse trials.

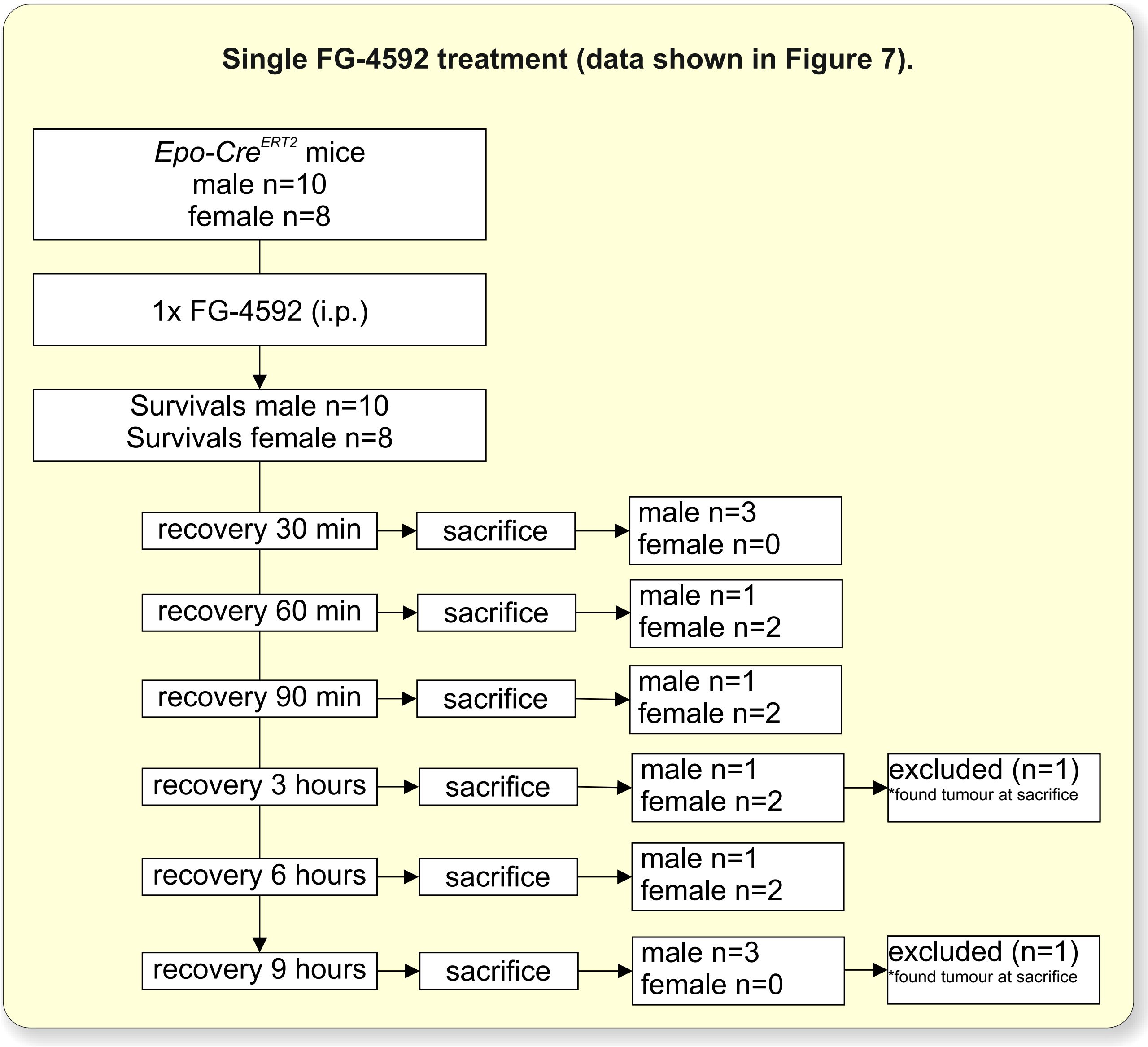

FIGURE S9 Flow diagrams of the mouse trials.

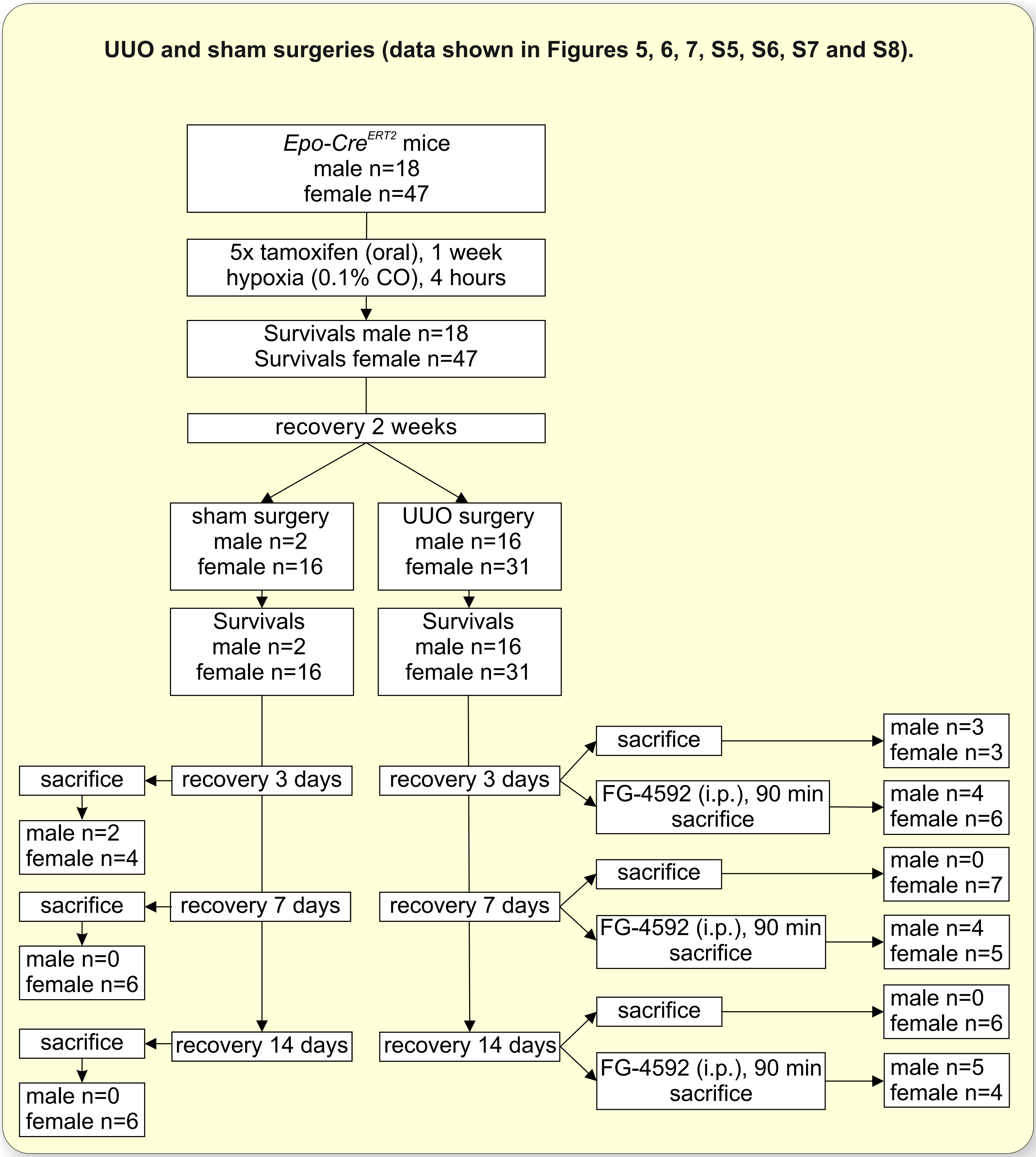

FIGURE S9 Flow diagrams of the mouse trials.
